# Information flow in first-order potts model phase transition

**DOI:** 10.1038/s41598-022-17359-w

**Published:** 2022-09-07

**Authors:** Joshua M. Brown, Terry Bossomaier, Lionel Barnett

**Affiliations:** 1 School of Computing & Mathematics, Charles Sturt University, Bathurst, NSW Australia; 2 Centre for Research in Complex Systems, Charles Sturt University, Bathurst, NSW Australia; 3 Sackler Centre for Consciousness Science, Department of Informatics, University of Sussex, Brighton, UK

**Keywords:** Information theory and computation, Phase transitions and critical phenomena

## Abstract

Phase transitions abound in nature and society, and, from species extinction to stock market collapse, their prediction is of widespread importance. In earlier work we showed that Global Transfer Entropy, a general measure of information flow, was found to peaks away from the transition on the disordered side for the Ising model, a canonical second order transition. Here we show that (a) global transfer entropy also peaks on the disordered side of the transition of finite first order transitions, such as ecology dynamics on coral reefs, which have latent heat and no correlation length divergence, and (b) analysis of information flow across state boundaries unifies both transition orders. We obtain the first information-theoretic result for the high-order Potts model and the first demonstration of early warning of a first order transition. The unexpected earlier finding that global transfer entropy peaks on the disordered side of a transition is also found for finite first order systems, albeit not in the thermodynamic limit. By noting that the interface length of clusters in each phase is the dominant region of information flow, we unify the information theoretic behaviour of first and second order transitions.

## Introduction

Numerous mechanisms for predicting phase transitions exist, applied, for example, from core science and engineering through biology, ecology, medicine and finance^1^: increased variance; critical slowing down^1^; flickering^2^; and a peak in the global transfer entropy (Eq. 2). Two canonical models of equilibrium transitions stand out: the Ising model^3^, a binary spin system on a square lattice, where each point on the lattice has a spin, which may point up or down; and the Potts model, which generalises Ising to spins with an arbitrary number of states, *q*, and reduces to the Ising model for $q=2$.

In the Ising model^3^, mutual information peaks *at* the transition between ordered and disordered phases^4,5^, as does *pairwise* transfer entropy^6^ (Eq. 4, Suppl. Material), while *global* transfer entropy (Eq. 2) peaks distinctly on the disordered side^7^. Here we extend this prior work on global transfer entropy to the *q*-state Potts model^8^ which exhibits first-order phase transitions for $q>4$^9^ *and provide a unifying framework for both transitions* (See Suppl. Material for details regarding mutual information in the first-order Potts model). At $q=5$ the transition is *weakly first order*^10^, implying a long correlation length and low latent heat. As *q* increases the correlation length decreases and the latent heat increases. We show that as the system becomes more strongly first order (i.e., $q>7$^11,12^) the behaviour of global transfer entropy, ${{\mathbf {G}}}$, diverges from the second-order behaviour: In the thermodynamic limit, ${{\mathbf {G}}}$ becomes discontinuous at the transition temperature, $T_c$, peaking at $T_c^+$.

The standard Potts model comprises a two-dimensional lattice of spins with periodic boundary conditions and size $N=L\times L$, where the system state is ${\mathbf {s}}=s_1,\ldots ,s_N$, with $s_i\in \{1,\ldots ,q\}$. The interaction energy between two neighbouring sites is $E_{ij}=-J\delta (s_i,s_j)$ giving the Hamiltonian ${\mathcal {H}} = -J\sum _{\langle i,j \rangle }\delta (s_i, s_j)$, where interaction strength $J=1$, $\delta (x,y)$ is the Kronecker delta function which is one if $x=y$ and zero otherwise, and $\langle i,j \rangle$ are all interacting pairs of sites in the system. Local site energy, $E_i$, is defined similarly, fixing *i* and summing over its four neighbours. The system is updated using Glauber dynamics^13^.

Overall alignment of the lattice is measured by its *magnetisation*, $M = (q \cdot S_{m} \langle s_{m} \rangle  - 1)/(q - 1)$^10^, where $S_m = \sum \delta (s_m, s_i) / N$ is the proportion of the dominant, *mode*, state, $s_m$, over all sites for the given configuration of the lattice, with $S_m$ ranging from $q^{-1}$ when all *q* states are equally represented to 1 where only one state is present in the lattice, giving magnetisation in the range [0, 1]. *M* serves as the order parameter and the order-disorder transition occurs—for $q=2$ and $q\ge 4$—at an intermediate temperature^14^

1 $$\begin{aligned} T_c = \Big [\log (1+\sqrt{q})\Big ]^{-1}\,, \end{aligned}$$

where the (thermodynamic) system is disordered ($M=0$) at temperatures above $T_c$ and non-zero below $T_c$. The behaviour at $T_c$ defines the transition order, where $q\le 4$ has continuous *M* (and discontinuous *dM*/*dT*) giving a second-order phase transition.

Transfer entropy, ${{\mathbf {T}}}$, measures (Eqs. 2, 4, Suppl. Material) information flow from one stochastic process, *Y*, to another, *X*—in this case the states of two neighbouring spins over time. Global transfer entropy measures the average information flow of the entire system to individual spin sites:

2 $$\begin{aligned} {{\mathbf {G}}}= \frac{1}{N} \sum _i {{\mathbf {T}}}_{{{\mathbf {s}}}\rightarrow {s_i}}\,. \end{aligned}$$

We note however, that all information—no matter its origin in the lattice—must flow to $s_i$ via its neighbours or its own past, and thus consider only the immediate neighbourhood of each site (including $s_i$) rather than ${\mathbf {s}}$ in Eq. (2)^7^. As with ${{\mathbf {T}}}$, ${{\mathbf {G}}}\ge 0$ with ${{\mathbf {G}}}=0$ iff each site $s_i$, conditioned on its past, is independent of its neighbours.

## Methods

Direct simulation of the higher order Potts models is not straightforward, since the first-order transition shows a void region of energy space around the phase transition, such that general purpose update schemes, such as Glauber dynamics, are very unlikely to enter this region. In fact, for temperatures close to the critical temperature, the energy distribution *P*(*E*) is bimodal (See Suppl. Material, Fig. S1). Thus we estimate ${{\mathbf {G}}}$ via two methods.

The first, denoted ${{\mathbf {G}}}^{(g)}$, employs straight-forward Glauber dynamics where each update, or *sweep*, comprises *N* spin flip attempts. The second uses the *density of states*, *d*(*E*), calculated with the Wang-Landau algorithm^15^ (See Suppl. Material). *P*(*E*) may then be calculated from

3 $$\begin{aligned} P(E) = d(E)\exp (-E/[k_bT])\,, \end{aligned}$$

where *E* is the lattice energy. Any thermodynamic observable, *f*(*T*), may now be determined from its value as a function of *f*(*E*)^16^

4 $$\begin{aligned} f(T) = \frac{\displaystyle \sum _E f(E) P'(E)}{\displaystyle \sum _E P'(E)}\,, \end{aligned}$$

where $P'(E)$ is the distribution of energies, and has been rescaled for visualisation and computational reasons (Specifically, normalisation is such that $P'(E) = \exp \Big [\log [gd(E)] - E/[k_bT] - \max (\log [d(E)] - E/[k_bT])\Big ]$. As the new term, $\max (\log [d(E)] - E/[k_bT])$, is constant over the summation, it cancels out such that *f*(*T*) is unmodified.).

After determining *d*(*E*) we need to determine ${{\mathbf {G}}}(E)$. While *f*(*E*) depends on energy only, ${{\mathbf {G}}}$ is a temporal quantity (Eq. 4, Suppl. Material), that is, it is a function of the change in state between $t-1$ and *t*, and thus also depends on temperature, therefore we in fact need to determine ${{\mathbf {G}}}(E,T)$ for varying *T*. Additionally, as $P(E) \rightarrow 0$ for many values, *f*(*E*) can be measured more simply by culling energy values where *P*(*E*) is sufficiently low—that is, once *d*(*E*) has been determined, and we move to determining ${{\mathbf {G}}}(E,T)$, reaching *every* *E* is unnecessary and so ${{\mathbf {G}}}(E,T)$ can be calculated via a separate set of realisations utilising only Glauber dynamics rather than Wang-Landau updating.

We thus collect ensemble statistics similar to ${{\mathbf {G}}}^{(g)}$ (with fixed *T* per ensemble), collating statistics for ${{\mathbf {G}}}(E,T)$ using the energy *E* of the lattice *before* the Glauber sweep, where the future state is the post-sweep state. We denote this regime as ${{\mathbf {G}}}^{(s)}$. We note however that this may not be strictly correct, as *E* can change after each successful spin flip during the sweep, thus statistics collated for ${{\mathbf {G}}}(E,T)$ will include elements from $E'\ne E$. To address this, we separately collate statistics on a per-flip basis, where $s_i$ and its neighbourhood are recorded at any attempt to flip $s_i$, giving ${{\mathbf {G}}}^{(f)}$.

Straight-forward Glauber approaches construct ensembles comprising $r=10$ realisations pooled together, with settling time of 1000 time steps, followed by a measurement sequence of $10^5$ time steps as in the Ising model^7^. Standard error calculated by generating 10 ensembles. We optimise simulation by modifying initialisation dependent on *T*. In the ordered regime, $T<T_c$, we initialise all realisations to the same ground state, noting that $T_c(L) > T_c$ [p. 4]^17^ and thus only the ordered peak exists in *P*(*E*) for $T<T_c$. For $T\ge T_c$ we evenly divide realisations into random ground states or random disordered states to sample both *P*(*E*) peaks. Density of states approaches constructed likewise, minus the superflous (in this regime only) settling time.

Due to data volume requirements involved in estimation of ${{\mathbf {G}}}$, ${{\mathbf {G}}}^{(s)}$ and ${{\mathbf {G}}}^{(f)}$ we employ a compression regime (explained below in the methodology). This regime is validated by applying it to ${{\mathbf {G}}}^{(g)}$, giving ${{\mathbf {G}}}^{(e)}$ (shown in the Suppl. Materials).

## Results and discussion

**Figure 1:**
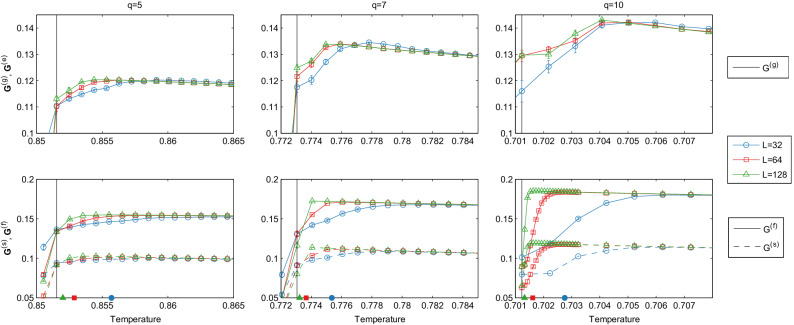
${{\mathbf {G}}}$ measured using three methods (top: ${{\mathbf {G}}}^{(g)}$, bottom: ${{\mathbf {G}}}^{(f)}, {{\mathbf {G}}}^{(s)}$), with $q=5,7,10$ (columns) for $L=32,64,128$. Ensemble collated using $10^5$ time steps over 10 realisations. Vertical lines indicate $T_c$. Filled symbols indicate “*effective*” $T_c(L)$, the location where *P*(*E*) is precisely bimodal for given *q*, *L*, corresponding to values found in analytical methods^11^ (see Suppl. Materials). Error bars calculated from 10 ensembles and are smaller than symbols in some regions. Gap between ${{\mathbf {G}}}^{(s)}$ and ${{\mathbf {G}}}^{(f)}$ due to extraneous data included in ${{\mathbf {G}}}^{(s)}$ (See main text).

All variants exhibit a peak in ${{\mathbf {G}}}$ on the disordered side of the transition (Fig. 1), with per-sweep versus per-flip statistics differing by a roughly constant factor: the statistics collated for ${{\mathbf {G}}}^{(s)}$ can be considered equivalent to those collated for ${{\mathbf {G}}}^{(f)}$ with a small amount of random noise applied, thus reducing the information flow and therefore ${{\mathbf {G}}}^{(s)}= c{{\mathbf {G}}}^{(f)}$, with $0<c<1$. The peak locations are mostly stable for the pure Glauber approach, ${{\mathbf {G}}}^{(g)}$, except for $L=32$, while the thermodynamic, density of states approaches, ${{\mathbf {G}}}^{(f)}, {{\mathbf {G}}}^{(s)}$, exhibit a strong shift in ${{\mathbf {G}}}$ peak as *q* and lattice sizes increase, rapidly approaching the critical temperature.

The behaviour of the ${{\mathbf {G}}}$ peak in ${{\mathbf {G}}}^{(g)}$ compared with the density of states approaches highlights the core issue with initialisation regimes and hysteresis. Specifically, for $T>T_c$ the current initialisation regime initialises half of the realisations in an ensemble to disordered and the other half to randomly chosen ground states, in an attempt to circumvent infeasibly long times to traverse the valley in *P*(*E*). This is done under the assumption that it achieves bimodal *P*(*E*) while rapidly collapsing to the correct unimodal *P*(*E*) on either side of $T_c(L)$. However, this collapse is not quick enough. For example, for $q=10$, the normalised ordered peak in *P*(*E*) should drop below $10^{-4}$ at $T \approx 0.7017$ (according to density of states estimation), however, in the Glauber simulation it does not reach this threshold until $T \approx 0.703$. That is, we have a spurious ordered peak above $T_c(L)$, resulting in an artificial reduction in ${{\mathbf {G}}}^{(g)}$ for $T_c(L)< T < {\sim }0.703$ (See Suppl. Materials). Here, by using Eq. (4) directly, as done here, we circumvent the issue altogether.

**Figure 2:**
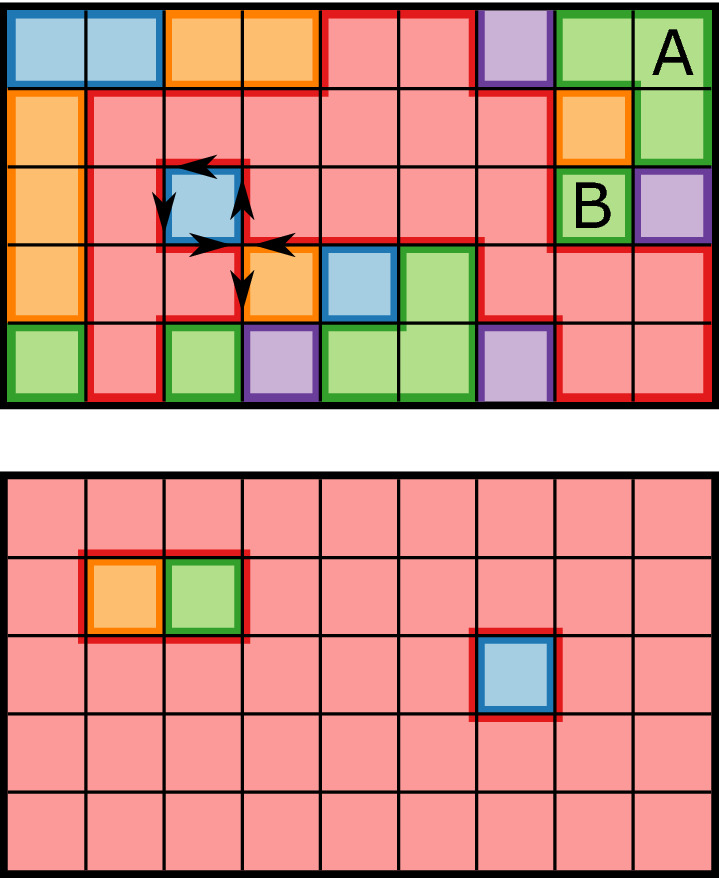
Interfaces for $q=5$ lattice sampled from $T=T_c$ (0.8515) (Top) and $T=0.5$ (Bottom) where each square is a lattice site. Top: Arrows show the counter-clockwise path interface walker (for large cluster) takes around complex interactions. Labelled clusters, while sharing the same state, are disjoint, and thus have separate interfaces. Average interface length is $(34 + 3\times 8 + 3 \times 6 + 9 \times 4)/16=7$. Bottom: When one cluster dominates, it no longer has an “outer” perimeter. Average interface length is $(6+4\times 4)/5=4.4$.

Finally, we look at a physical understanding of the behaviour of ${{\mathbf {G}}}$. Intuitively, information flows when neighbour states differ, hence zero information flow in ground states. This behaviour necessarily extends to clusters of states, implying information flow occurs on the boundaries, or interfaces, between clusters (See Fig. 2). It seems reasonable then to assume that information flow scales with number of interfaces. However, such a maximum coincides with the zero-energy fully disordered regime, where quite clearly ${{\mathbf {G}}}=0$. This assumption neglects the temporal nature of ${{\mathbf {G}}}$, which is disrupted at high temperature.

Remember that ${{\mathbf {G}}}$ is a measure of a site’s dependence on neighbouring sites, conditioned on its own past. At high temperature, spin flips are essentially random, choosing new states with little influence from neighbours. As temperature decreases, neighbour influence increases, leading to clusters of similar sites. We can thus approximate average influence by probability of cluster size, *p*(*c*). This influence is the manifestation of information flow in the system, but only along interfaces (since information flow is conditioned on its own past), leading to the conjecture:

5 $$\begin{aligned} {{\mathbf {G}}}\propto \sum _c p(c)L_c\,, \end{aligned}$$

where $L_c$ is the average interfacial length of cluster of size *c*. Note however that when clusters get sufficiently large—i.e., on the order of system size *L*—they no longer have an outer perimeter and are instead defined by the holes created by other clusters (Fig. 2, bottom). Thus for this dominant cluster to increase in size, the internal holes must shrink and its interfacial length $L_c$ actually falls. As temperature decreases, influence increases, but the available sites to transfer influence decreases, hence total information flow ${{\mathbf {G}}}$ falls.

We note that Eq. (5) is essentially the *average* interface length. There should thus be some relationship between average interfacial length and net information flow in the lattice.

The average interface length is defined as:

6 $$\begin{aligned} \langle I_l \rangle = \frac{\sum _x^{N_I} I_{(x,l)}}{N_I}\,, \end{aligned}$$

where $N_I$ interfacial lengths are found by performing a “*turn-right walk*” procedure, similar to Saberi^18^, on every unmarked edge between adjoining lattice sites of *differing* states. Edges are marked during the walk procedure in association with an adjoining site (such that each edge is ultimately left unmarked or marked twice). This prevents a cluster from counting its perimeter (of length $N_i$) $N_i$ separate times, but correctly accounts for interface boundaries between a cluster and all of its neighbouring clusters. This also addresses clusters with two or more disjoint interfaces, i.e., a 2D doughnut.

Interface results, *I*(*T*), are calculated from *I*(*E*) and Eq. (4) with the weighted Wang-Landau update scheme^15^. Each *E* value sampled at minimum 5000 times, up to a maximum of 10000 samples.

**Figure 3:**
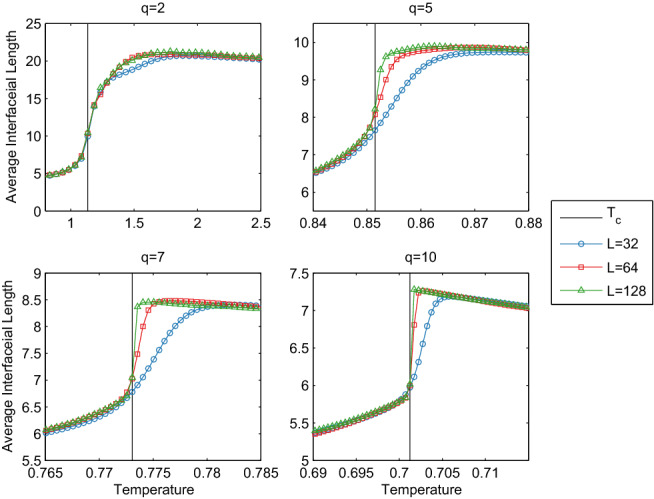
Average interface length for systems with $q\in \{2,5,7,10\}$ for indicated lattice sizes. The behaviour in peak location mimics the behaviour of the ${{\mathbf {G}}}$ peak in all systems: the first-order cases, $q\in \{5,7,10\}$, converge to $T_c$ as the system becomes more strongly first order (increased *q*, *L*), while the second-order peak $q=2$ remains stable above the phase transition. Note the factor of two difference in temperature for $q=2$ and Ising results is simply due to a slight difference in definition of site energy (i.e., $E_{ij}$), with no further side effects.

The intuitive interface model of Eqs. (5) and (6), shown in Fig. 3, gives a remarkably good match to the ${{\mathbf {G}}}$ trends, peaking in the disordered regime in all cases, and converging to $T_c$ only where systems become more strongly first-order (increased *q* and increased *L* for $q>4$). In the $q=2$ Ising case, interface peak location remains stable at increasing lattice sizes, as does ${{\mathbf {G}}}$ peak location^7^.

Given the excellent agreement between the average interface length and ${{\mathbf {G}}}$, the average interface length is a suitable theoretical justification for ${{\mathbf {G}}}$, fitting the behaviour for the first- and second-order transitions into a single unified framework. It is known that in—at least some class of—second-order phase transitions, that ${{\mathbf {G}}}$ can serve as a predictor for an impending phase transition in systems slowly approaching criticality from the disordered regime^7^, and we show here that the same behaviour is exhibited in the first-order Potts model. Specifically, detection of a peak in ${{\mathbf {G}}}$ while other information theoretic metrics^7^ continue to rise could indicate an imminent transition. This behaviour of falling GTE while other information theoretic quantities rise holds most strongly for finite-size systems, that is, those with most practical utility, while it’s possible that in at least the $q=10$ case above, ${{\mathbf {G}}}$ may converge to $T_c$ in the thermodynamic limit.

## Supplementary Information

Supplementary Information.
