# Supplementary material for: Information flow in first-order potts model phase transition
Source: Sci Rep. 2022 Sep 7;12:15145. doi: 10.1038/s41598-022-17359-w (PMC9452544; doi:10.1038/s41598-022-17359-w)
Supplement: Supplementary file 1 — Supplementary Information. [file 41598_2022_17359_MOESM1_ESM.pdf]

SUPPLEMENTARY MATERIAL

Glauber Dynamics

The system is updated using Glauber dynamics [1], where site s_i transitions to state s_k with probability

$$P(s_i \to s_k) = \left[1 + e^{\Delta E_{ki}/(k_b T)}\right]^{-1}, \quad (1)$$

where T is the system temperature, k_b is taken as one, and ΔE_{ki} denotes the difference in site (or system) energy should the flip occur—i.e., $\Delta E_{ki} = E_k - E_i$. This transition probability biases spin flips towards lower energy states—where the ground state occurs at minimum energy when all sites take the same state—while the system temperature inhibits this bias, which disappears as $T \to \infty$ such that spins flip to random states with probability 0.5. Glauber dynamics satisfy detailed balance [2] and thus yield the thermal equilibrium probabilities at stationarity.

Density of States

The Density of States, $d(E)$, for the Potts model is calculated using Wang-Landau algorithm [3]. This algorithm involves simulating single-spin-flip mechanics with a transition probability:

$$p(E_1 \to E_2) = \min\left(\frac{d(E_1)}{d(E_2)}, 1\right) \quad (2)$$

where $d(E)$ is updated by a modification factor $f > 1$ each time energy E is visited (i.e. $d(E) \to fd(E)$), and the histogram $H(E)$ is accumulated. Essentially, the algorithm takes a random walk through energy space. If an energy is encountered frequently, we want to decrease the chance that the spin configuration will be hit again, so we increase its energy—by factor f—making a transition to this state less likely. If we keep doing this, the high-probability states will get visited less and less often and the overall histogram of energy states for these modified energies will be approximately flat. The factor, f, by which we increase the energy determines how lumpy the histogram is. Once the histogram is sufficiently *flat*, H is reset and the modification factor is reduced ($f \to \sqrt{f}$), allow the new histogram to be less lumpy than previously. This then repeats until f is sufficiently small (i.e. $1 < f \leq \exp(10^{-8})$).

This requires every energy level E to be visited throughout the simulation, which becomes increasingly difficult as q and L are increased. To ensure enough energy space is explored, one can divide the energy space into subintervals, sampling each separately, but issues exist at subinterval boundaries. Another approach involves adaptive subintervals [4] which addresses these boundary issues. However, for the lattice sizes we studied, we were able to instead run the simulation longer to allow time for full traversal to occur. We performed L^2 spin-flip attempts per (random-)sweep of the lattice, for at least 10^4 sweeps. We then start checking the flatness of H, continuing to iterate until H is flat, that is, the minimum value seen in H is greater than or equal to 80% of the average value of H. We then reduced f and reset the histogram and began a new iteration of the simulation. Simulates took 27 such iterations to reach the desired final factor threshold, $f < \exp(10^{-8})$. Figure S2 shows this approach was sufficient to explore the full energy space for the most difficult configuration we tried—$q = 10$ and $L = 128$—allowing us to generate a bimodal $P(E)$. All parameter configurations explored were confirmed to reach such bimodal distribution (not shown).

		$T_c(L)$			T_c
		32	64	128	
	5	0.855557	0.852959	0.852023	0.851528
q	7	0.77541	0.773727	0.773268	0.773059
	10	0.702773	0.701635	0.701331	0.701232

Table I. Transition temperature, $T_c(L)$, for varying q, L along with the with the transition temperature of the thermodynamic system. Generated from the density of states distributions for each q, L pairing.

Effective T_c

$T_c(L)$ is estimated from the density of states, $d(E)$. It is known that for first-order systems, $P(E)$ is bimodal around the transition temperature [3]. We employ a binary search of 40 iterations to find the value of $T_c(L)$ such that peak height is equal (See Figure S2 (Center) for an example using $q = 10, L = 128$).

The values found for $T_c(L)$ at all q, L studied, as well as T_c, are presented in Table I. These values are used to place the filled symbols in Fig. 1 of the main text.

Mutual Information

Mutual Information and Transfer Entropy have both been applied to the Ising model [5–8]. This study explores the Global Transfer Entropy metric for the first-order Potts model, leaving only Mutual Information to be explored. This has been explored in Brown [9]. As Mutual Information is an instantaneous quantity, it can be calculated directly using Eqn. 4 from the main text—specifically, by utilising $P(E)$. Figure S1 shows the results from this prior study, showing that Mutual Information indeed converges to the transition temperature as the system approaches the thermodynamic limit.

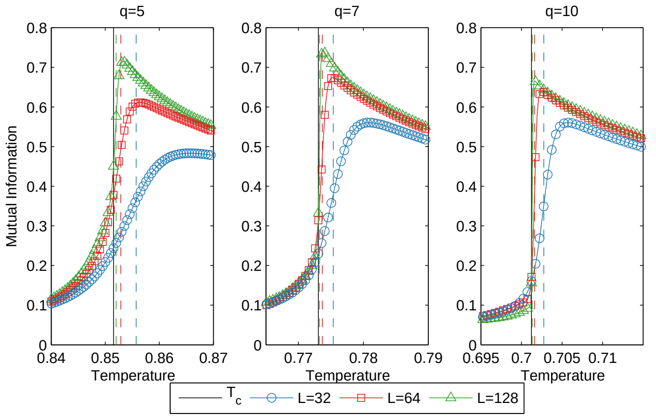

Figure S1. Mutual Information for q-state Potts model at varying lattice sizes, measured using Density of States approach following Wang and Landau [3]. Dashed vertical lines indicate effective critical temperature of lattice size, $T_c(L)$. Reprinted from Brown [9].

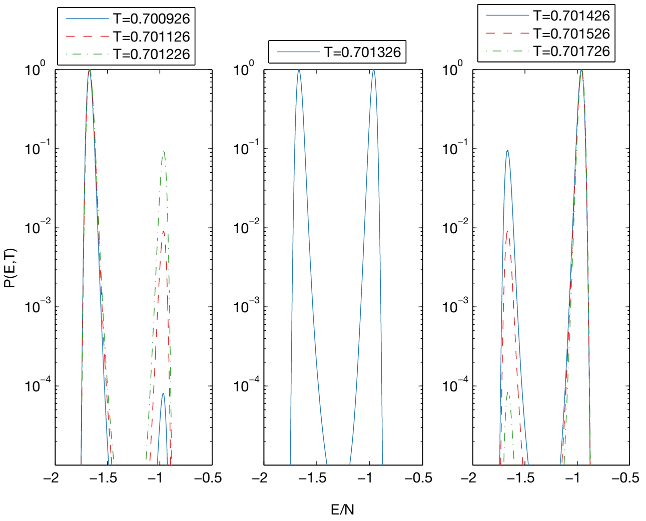

Figure S2. $P(E)$ of Potts states for $q = 10, L = 128$. Left: $P(E)$ as $T \to T_c^-$, demonstrating emergence of right (disordered) peak. Middle: The location of the "effective" transition, $T_c(L)$, defined by equal height peaks. Right: $P(E)$ as T moves away from $T_c(L)$, showing dissolution of ordered peak. Note that in the thermodynamic limit, each peak only exists in its relevant regime and emergence of bimodal peaks is instantaneous at T_c.

Transfer Entropy

Transfer entropy measures information flow from one stochastic process, Y, to another, X—in this case the states of two neighbouring spins over time. It is a non-negative quantity, reaching zero iff process X, conditioned on its own past, is independent of the past of Y. Positive values indicate a statistical dependency—a reduction in uncertainty—of X given knowledge of the past of Y. Transfer entropy is given by the time-lagged mutual information, conditioned on the past of X:

$$\mathbf{T}_{Y\to X} = \mathbf{I}(X_t : Y_{t-1} \mid X_{t-1}) \,, \quad (3)$$
$$= \mathbf{H}(X_t \mid X_{t-1}) - \mathbf{H}(X_t \mid X_{t-1}, Y_{t-1}) \,, \quad (4)$$

where we use a single-step time-lag and the pairwise transfer entropy is simply the average transfer entropy over all interacting sites:

$$\mathbf{T}_{pw} = \frac{1}{N} \sum_{\langle i,j \rangle} \mathbf{T}_{s_j \to s_i} \,. \quad (5)$$

Energy Space

The first-order transition shows a void region of energy space around the phase transition, such that general purpose update schemes, such as Glauber dynamics, are very unlikely to enter this region. In fact, for temperatures close to the critical temperature, energy distribution $P(E)$ is bimodal (See Fig. S2—note a scaling factor is introduced such that peak maximum is one). As q decreases, the peaks shift closer together until they merge into a unimodal peak at $q = 4$ (characteristic of a second-order transition). The valley between peaks is shallower for given lattice size at lower q, thus $q = 5$ is considered weakly first-order, while $q = 10$ is strongly first-order. As L increases (with constant q) the valley deepens, making simulation for large lattices, particularly at $q = 10$, increasingly difficult.

Neighbourhood Compression

$\mathbf{G}$ is estimated via plug-in entropy estimators, using histograms to determine distributions. Such a histogram requires six dimensions (a site, its four neighbours and its future) of q elements in each dimension—which requires q^{12} data points for effective estimation (using the heuristic $B = \sqrt{N}$ [10], where $B = q$ is the number of bins in each dimension of the histogram). This volume of data is difficult, yet achievable, for a single histogram (as in $\mathbf{G}^{(g)}$), but completely infeasible for E histograms, as required in $\mathbf{G}^{(s)}$ and $\mathbf{G}^{(f)}$. Thus we require some way to compress the histogram.

To accomplish this, we note that the transition probability depends only upon the number of spins matching the initial and final spins, rather than the exact neighbouring states. An intuitive approach replaces the neighbour dimensions with the energy delta term, ΔE_{ki}, as this should encode all transition information. This approach is incorrect however as it incorporates information about the future state directly into the conditioned variables in the second term of Eqn. 4—that is, Y_{t-1} incorrectly

becomes some function of X_t.

Thus we encode just the current site energy, E_i, although not without trade-off: removal of neighbour details leads to consistent reduction in total available information (See Fig. S3, top row). This approach is validated with an alternative reduction with consistent results—where the binary function, $\delta(s_i, s_j)$, is used for each neighbour. These approaches give significant reductions in data requirements—$(5q^2)^2$ and $(2^4q^2)^2$ respectively. The former approach will be employed as it requires fewer bins, and thus data points, without effect on the result.

Lattice Initialisation

The main text describes an artificial reduction in the magnitude of $\mathbf{G}^{(g)}$ across all values of T for the experimental configuration when compared to the density of states approaches. This reduction can be partially abated by increasing the ensemble size with more realisations of the Glauber simulations, however we observe diminishing returns, requiring too many computational resources to sufficiently eliminate the reduction. Observation of the distribution of energy states from these realisations highlights the unavoidable weakness of simulation based approaches: infeasible simulation time. The initialisation regime employed is intended to side-step this weakness and produce bimodal $P(E)$: realisations are evenly initialised to disordered and ordered states at $t = 0$ for $T \geq T_c$ under the assumption that as temperature increases, the ordered realisations will rapidly dissolve into disorder. This would then circumvent issues with traversing the valley in $P(E)$. However, observation reveals that the high-temperature dissolution does not occur rapidly enough: the normalised ordered peak is above 10^{-4} until approximately $T = 0.703$, where for $q = 10$ the density of states estimation shows the peak should drop below 10^{-4} at $T \approx 0.7017$.

If instead realisations at $T > T_c$ are initialised simply to disordered states, thus avoiding spurious ordered peaks in $P(E)$, then $\mathbf{G}^{(g)}, \mathbf{G}^{(e)}$ increase monotonically approaching T_c, past $T_c(L)$, as seen in Fig. S4. This highlights the same problem from a different angle: impractically large observation windows are required to traverse the valley, and no bimodal peak is obtained at all at $T_c(L)$ (i.e., a spurious *lack* of ordered peak) which artificially inflates $\mathbf{G}$. Thus simulation approaches are inappropriate for determining the limiting behaviour of $\mathbf{G}$ near the transition, as they require infeasible simulation time or finely tuned initialisation regimes, noting the latter is only possible given $P(E)$. Yet this is redundant: if one has $P(E)$ then Eqn. 4 (main text) can be utilised directly, as we have done, thus circumventing these issues altogether. $\mathbf{G}^{(f)}$ and $\mathbf{G}^{(s)}$ have been included in Fig. S4, noting that $\mathbf{G}^{(s)}$ produces coincident results with the disorder-initialised $\mathbf{G}^{(e)}$ away from the transition, demonstrating the equivalence of the two approaches where the system is unimodal and disordered (and thus initialisation issues are moot).

Similar hysteresis issues arise for $T < T_c$. Figure S5 shows $\mathbf{G}^{(e)}$ and magnetisation with varying proportion of lattice elements, α, initialised to the $s_i = 0$ state. When all, or most, states are randomly initialised ($\alpha < 0.25$), no single state is able to dominate the entire lattice at the temperatures simulated, resulting in $M < 0.6$, and very low M near T_c. As α increases, nucleation sites become stable enough at low T to begin to dominate the lattice, however still remains low near T_c. Finally as $\alpha = 0.5$, the $s_i = 0$ ground state nuclei remain stable, resulting in high M. Note that it is only when M approaches 1 (and thus $s_i = 0$ begins to dominate), does $\mathbf{G}^{(e)}$ drop significantly, with all $\mathbf{G}^{(e)}$ values peaking around the approximate inflection point of the magnetisation.

Limiting Behaviour

The limiting behaviour of $\mathbf{G}$ can be determined via closer analysis of $\mathbf{G}(E, T)$. Figure S6 shows $\mathbf{G}^{(f)}(E, T)$ and $P(E)$ at $q = 10, L = 128$ for selected temperatures. As with the above regimes, realisations are initialised evenly between random ground states and disordered states. We can observe the valley in $\mathbf{G}^{(f)}(E, T)$ which realisations are unable to traverse, noting that for $T \approx 0.702, 0.707$, while no ordered $P(E)$ peak exists, $\mathbf{G}^{(f)}(E, T)$ is non-zero due to initialisation regime. As T increases, the system is able to move through this region of energy space, until high enough temperatures are reached such that lower energies become impossible, with the ground state realisations very rapidly becoming disordered.

Consider now the extreme energies (effectively temperatures) in Fig. S6. On the disordered end ($E/N = -1$), we can see that $\mathbf{G}^{(f)}(E, T)$ peaks below the disordered $P(E)$ peaks, and steadily decreases at higher energies (and thus temperatures), consistent with the expectation of reduced $\mathbf{G}$ as spins become increasingly independent. Similarly, as $T \to 0$, low energy $\mathbf{G}^{(f)}(E, T)$ goes to zero as well: conditioned on its own past, s_i becomes independent of its neighbourhood—the neighbourhood adds no additional information to knowing the past of s_i—as expected.

The observation of high energy $\mathbf{G}^{(f)}(E, T)$ peaking earlier than $P(E)$, in the void region, also resolves the limiting behaviour near T_c. Specifically, when moving towards T_c (and thus $P(E)$ peaks at progressively lower E/N) from high temperatures $\mathbf{G}^{(f)}(E, T)$ is always increasing. Therefore in the thermodynamic limit, where $P(E)$ is unimodal until precisely T_c, $\mathbf{G}$ will increase towards T_c. The peaks appearing in Fig. 1 (main text) away from T_c are then due to the finite size effect of

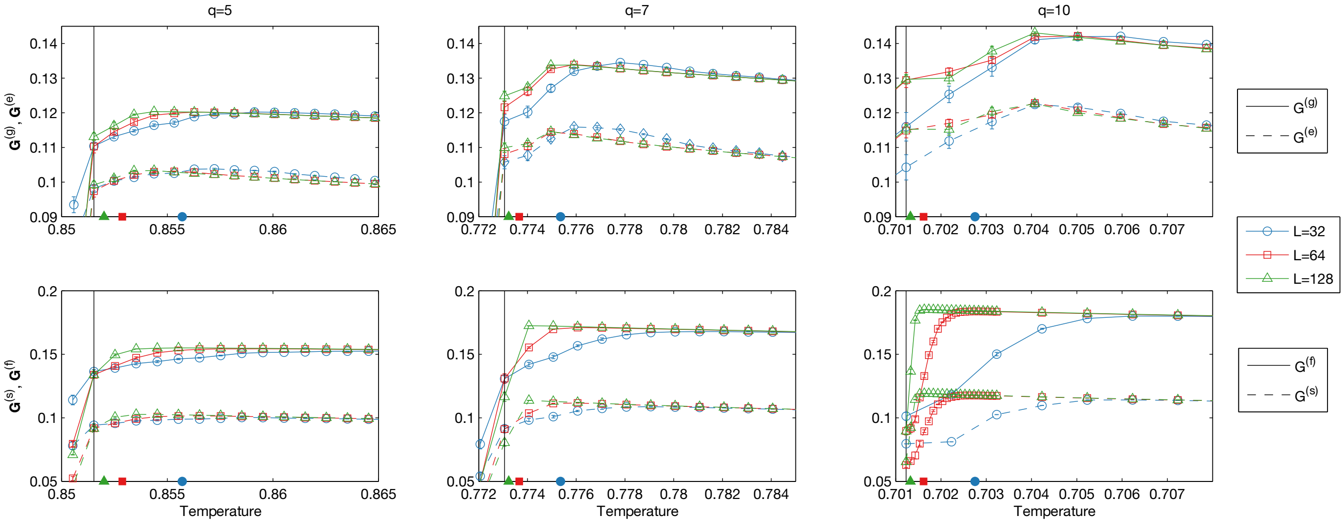

Figure S3. Fig. 1 (main text) repeated with validation from $\mathbf{G}^{(e)}$. Note that $\mathbf{G}^{(e)}$ tracks $\mathbf{G}^{(g)}$ with a constant reduction due to information lost in the compression algorithm.

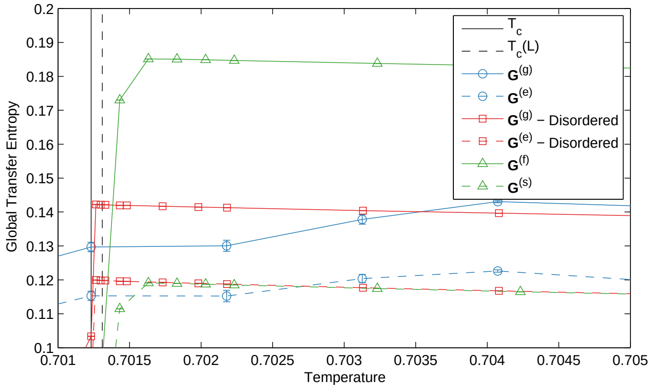

Figure S4. $\mathbf{G}^{(g)}$ for $q = 10, L = 128$ calculated over 10^5 time steps with 10 realisations per ensemble demonstrating two initialisation regimes for $T > T_c$: 1) half of the realisations initialised to randomly chosen ground states and half disordered against 2) all realisations initialised to disorder. Both regimes use the bimodal initialisation for $T = T_c$. $\mathbf{G}^{(f)}, \mathbf{G}^{(s)}$ also included. Note that while the disorder-initialised $\mathbf{G}^{(e)}$ is coincident with $\mathbf{G}^{(f)}$ away from T_c, it does not exhibit the same drop at $T_c(L)$ which should occur due to the appearance of bimodal $P(E)$.

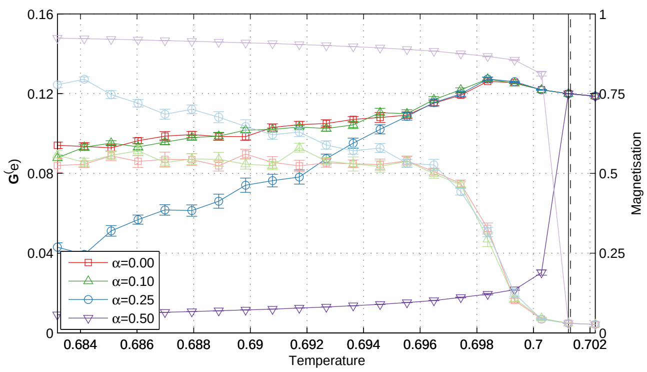

Figure S5. $\mathbf{G}^{(e)}$ (dark lines) and M (light lines) for $q = 10, L = 128$ calculated over 10^5 time steps with 10 realisations per ensemble with varying proportions, α, of elements initialised to $s_i = 0$, demonstrating hysteresis effects for $T < T_c$.

bimodal $P(E)$ away from T_c where low $\mathbf{G}$ ordered regimes are incorrectly sampled. Furthermore, in the limit at T_c, a system will be either ordered or disordered with valley $P(E) = 0$, and consequently $\mathbf{G}$ will be undefined at T_c.

[1] R. J. Glauber, Time-dependent statistics of the ising model, Journal of Mathematical Physics **4**, 294 (1963).

[2] N. G. Van Kampen, *Stochastic Processes in Physics and Chemistry*, Vol. 1 (Elsevier, 1992).

[3] F. Wang and D. Landau, Determining the density of states for classical statistical models: A random walk algorithm to produce a flat histogram, Physical Review E **64**, 056101 (2001).

[4] A. G. d. Cunha-Netto, A. Caparica, S.-H. Tsai, R. Dickman, and D. P. Landau, Improving wang-landau sampling with adaptive windows, Physical Review E **78**, 055701 (2008).

[5] H. Matsuda, K. Kudo, R. Nakamura, O. Yamakawa, and T. Murata, Mutual information of Ising systems, International Journal of Theoretical Physics **35**, 839 (1996).

[6] H. W. Lau and P. Grassberger, Information theoretic aspects of the two-dimensional ising model, Phys. Rev. E **87**, 022128 (2013).

[7] T. Schreiber, Measuring information transfer, Physical Review Letters **85**, 461 (2000).

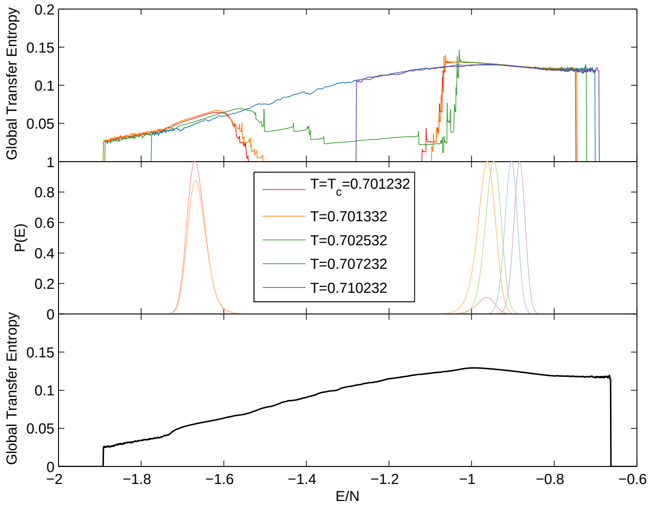

Figure S6. $\mathbf{G}^{(f)}(E,T)$ (top) for selected temperatures at and above T_c with $P(E)$ (middle) for $q=10, L=128$ with 10 realisations, half initialised to random ground states and half to disordered states. For temperatures near T_c, we observe a valley in $\mathbf{G}^{(f)}(E,T)$ as in $P(E)$, where realisations are unable to traverse. As T increases, central energy values are reachable, with lower energy values becoming unreachable—note that $\mathbf{G}^{(f)}(E,T)$ drops to zero at $E/N \approx -1.75, -1.3$ for $T \approx 0.707, 0.710$, respectively. Bottom shows $\mathbf{G}^{(f)}(E,T)$ average over T where each $\mathbf{G}^{(f)}(E)$ is scaled with respect to frequency over E.

[8] L. Barnett, M. Harré, J. Lizier, A. K. Seth, and T. Bossomaier, Information flow in a kinetic Ising model peaks in the disordered phase, Physical Review Letters **111**, 177203 (2013).

[9] J. Brown, *Information Theoretic Measures of Transitions to Collective Behaviour*, Ph.D. thesis, Charles Sturt University Australia (2018).

[10] W. N. Venables and B. D. Ripley, *Modern Applied Statistics with S* (Springer New York, 2002).
